# Molecular identification of sarcocysts from tissue of fallow deer (*Dama dama*) farmed in the open pasture system based on ssu rRNA gene

**DOI:** 10.2478/s11686-019-00159-0

**Published:** 2020-01-24

**Authors:** Władysław Cabaj, Justyna Bień-Kalinowska, Katarzyna Goździk, Katarzyna Basałaj, Żaneta Steiner-Bogdaszewska, Marek Bogdaszewski, Bożena Moskwa

**Affiliations:** Witold Stefański Institute of Parasitology of the Polish Academy of Sciences, Twarda 51/55, 00-818 Warszawa, Poland

**Keywords:** Fallow deer, *Sarcocystis* spp., Tissue cysts, Sequencing

## Abstract

**Purpose:**

*Sarcocystis* spp. are protozoan parasites of livestock which also infect birds, lower vertebrates and mammals, including man. Wild and domestic ruminants such as red deer, roe deer, fallow deer, cattle, sheep and goats may act as intermediate hosts for many *Sarcocystis* species, some of which are significant pathogens causing sarcocystosis in livestock and humans. The purpose of the present study was to determine the prevalence of *Sarcocystis* species in fallow deer farmed in an open pasture system.

**Methods:**

Samples of heart and oesophagus tissue taken from five fallow deer were examined by light microscope for the presence of sarcocysts. Genomic DNA was extracted from individual sarcocysts. ssu rRNA was successfully amplified using their DNA as templates.

**Results:**

Analysis of the ssu rRNA identified the presence of two *S. morae* sarcocysts in the heart tissue; similarly, *S. gracilis* sarcocysts were identified in the heart and oesophagus, and *Sarcocystis* sp. most closely related to *S. linearis* and *S. taeniata* were detected in oseophagus.

**Conclusions:**

These findings confirm the presence of *Sarcocystis* spp. in farmed fallow deer in Poland; however, more molecular studies are needed.

## Introduction

*Sarcocystis* spp. are obligate heteroxenous protozoan parasites (Apicomplexa, Sarcocystidae): the continuation of their life cycle requires the presence of both intermediate and definitive hosts. The intermediate host, which is usually a herbivore or omnivore infected by ingesting food or water contaminated with sporocysts, hosts the asexual phase of the life cycle. In immature cysts, further asexual reproduction takes place by repeated endodyogeny of metrocytes. Mature cysts contain several hundred thousand bradyzoites; these do not divide further and represent the terminal asexual stage in the intermediate host. Following the ingestion of their cysts by a carnivorous definitive host, the bradyzoites initiate the sexual phase of the life cycle (gamogony) in the cells of the small intestine of the host, ultimately resulting in the formation of oocysts [1]. Free sporocysts are released into the intestinal lumen and passed into the soil or water of the surrounding environment with the faeces.

Sarcocystosis is a zoonotic disease presented by a wide range of domestic ungulates, including cattle, sheep, goats or water buffalo, or wild ones, such as camels and wild boar [2-6]. The *Sarcocystis* of cervids has been intensively studied over the last few years. At least 14 *Sarcocystis* species have so far been identified in red deer (*Cervus elaphus*) in Europe: *S. cervicanis, S. elongata, S.* cf*. grueneri, S. hardangeri, S. hjorti, S.* cf*. hofmanni, S. iberica, S. linearis, S. morae, S. ovalis, S. taeniata, S. tarandi, S. truncata* and *S. venatoria* [3, 4, 7, 8]; however, little is known of their potential zoonotic or public health significance [9, 10]. Although more than 200 valid *Sarcocystis* species have been identified, only three, *S. hominis, S. suihominis*, and *S. heydorni*, have so far been shown to be capable of causing intestinal disease in humans [4, 11]. Although the intestinal forms of infection are often asymptomatic and self-limiting, a number of studies have reported symptoms ranging from mild gastrointestinal distress to nausea, loss of appetite, vomiting, stomach ache, bloat, diarrhea, dyspnea and tachycardia [12]. Whether individuals remain asymptomatic or develop some degree of disease appears to be related to the quantity of the meat consumed, inoculum size and various host factors [12]. Compared to red deer, less data concern on fallow deer (*Dama dama*) [13-18]. The latest work [19] report fallow deer in Spain as a new host for *S. morae*, originally described from the red deer. Despite being regularly consumed in other parts of the EU, meat from fallow deer and red deer is just entering the market for human consumption in Poland. It is important to note that although the numbers of wild and farmed fallow deer have increased over the last decade in the country, no studies have yet examined the presence of *Sarcocystis* species among them. The present paper, therefore, aims to determine the presence of *Sarcocystis* species in fallow deer farmed in an open pasture system.

## Materials and methods

### Animals and sampling

The study was carried out in the Breeding Station of the Witold Stefański Institute of Parasitology, Academy of Sciences in Kosewo Górne (Region of Warmia and Mazury; Poland; N: 53° 48′; E: 21° 71 23′). Samples of heart and oesophagus tissue were obtained from five 1-year-old male fallow deer slaughtered for commercial purposes. All samples (10 g of weight) were gently broken up in physiological saline solution using a blender and filtered through gauze and the resulting sediments were collected. The basic morphology (shape, size) of any cysts present was examined in wet mounts under an inverted Olympus IX50 microscope fitted with a camera. The samples were preserved for molecular investigation in sterile H_2_O in Eppendorf tubes and stored at − 72 °C.

### DNA extraction

Individual sarcocysts were detected in muscles by light microscopy. Total DNA was extracted from 10 individual sarcocysts using the NucleoSpin®Tissue kit (Macherey–Nagel, Germany) according to the manufacturers’ instructions. The DNA was eluted in 50 μl of distilled water. The extracted DNA was stored at − 20 °C for PCR assay.

### Two different PCR protocols were employed for the molecular examination of tissue cysts

In the first protocol, DNA amplification was performed using PCR targeted at a 18S rDNA sequence of approximately 900 bp in accordance with More et al. [20]. The following specific primers were used: SarcoFext (GGT GAT TCA TAG TAA CCG AAC G) and SarcoRext (GAT TTC TCA TAA GGT GCA GGA G).

Reactions were performed in a final volume of 25 µl reaction mixture with 1 unit of Taq DNA polymerase per reaction (Thermo Fisher Scientific) under the following conditions (final concentrations): 1× reaction buffer supplied with the DNA polymerase, 1.25 mM MgCl_2_, 200 mM dNTPs, 0.2 M of each primer and the following cycler program: initial denaturation (95 °C, 5 min), followed by 40 cycles of denaturation (94 °C, 40 s), annealing (59 °C, 1 min), elongation (72 °C, 1 min) and a final extension at 72 °C (10 min).

The PCR products were analyzed by electrophoresis in 1.5% agarose gel and stained with GelRed (Nucleic Acid Gel Stain, Biotium). PCR products 900 bp in size were cut out from the gel and purified using Clean-up Product Purification Kits (A&A Biotechnology, Poland) according to the manufacturer’s instructions. DNA concentration was estimated using a NanoDrop ND-1000 Spectrophotometer (NanoDrop Technologies, USA).

The PCR amplicons were then ligated into pGEM-T easy cloning vector (Promega). *Escherichia coli* strain XL-1 Blue MRF electrocompetent cells (Promega) were used for cloning. Positive clones (six) were identified by colony PCR with primers directed against vector sequences outside the multi-cloning site. The clones found to contain inserts were used for further examination. Positive plasmids were purified using GeneAll Exprep Plasmid SV mini (GeneAll, Korea) according to the manufacturer’s instructions.

The ssu rRNa gene products concentration was measured using a NanoDrop ND-1000 Spectrophotometer (NanoDrop Technologies, USA) and was then sequenced (Genomed, Poland). The sequence information obtained from all the isolated clones was assembled using Vector NTI Advance 10 software (Invitrogen, Scotland). The complete sequences were checked against sequences published in GenBank using BLAST (https://www.ncbi.nlm.nih.gov/BLAST/).

In the second protocol, the ssu rRNA gene was amplified by PCR using the primers for *Sarcocystis* [ERIB1 5′-ACC TGG TTG ATC CTG CCA G-3′, Primer1L 5′-CCA TGC ATG TCT AAG TAT AAG C-3′, Primer3H 5′-GGC AAA TGC TTT CGC AGT AG-3′] [21] in a 25 μl final reaction volume with one unit of Taq DNA polymerase/reaction (Thermo Fisher Scientific). The following final concentrations of reagents were used: 1× reaction buffer, 1.5 mM MgCl_2_, 250 μM dNTPs, 0.5 μM of each primer. PCR was started with initial denaturation (95 °C, 5 min), followed by 35 cycles of denaturation (95 °C, 30 s), annealing (55.5 °C, 30 s), elongation (72 °C, 1 min) and a final extension at 72 °C (10 min).

PCR products were analyzed and sequenced as described above. The purified products were then sequenced (Genomed, Poland).

### Phylogenetic analyses

The evolutionary history was inferred using the Maximum Likelihood method based on the Kimura 2-parameter model [22]. The tree with the highest log likelihood (− 2638.89) is shown. The percentage of trees in which the associated taxa clustered together is shown next to the branches. Initial tree(s) for the heuristic search were obtained by applying the Neighbor-Joining method to a matrix of pairwise distances estimated using the Maximum Composite Likelihood (MCL) approach. A discrete Gamma distribution was used to model evolutionary rate differences among sites [four categories (+G, parameter = 0.3385)]. The tree is drawn to scale, with branch lengths measured in the number of substitutions per site. The analysis involved 100 nucleotide sequences. All positions with less than 95% site coverage were eliminated. That is, fewer than 5% alignment gaps, missing data, and ambiguous bases were allowed at any position. There were a total of 789 positions in the final dataset. Evolutionary analyses were conducted in MEGA7 [23].

## Results

**Fig. 1:**
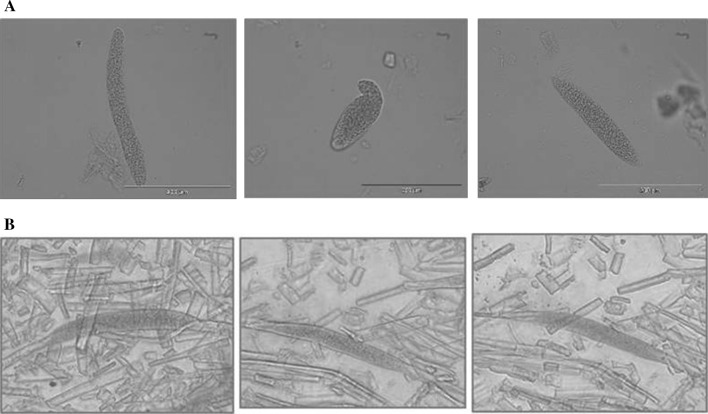
The appearance of unstained sarcocysts isolated from fallow deer in Kosewo, Poland, under the light microscope: lens 10 × 25PhC; eyepiece 10× WH10 × 22; magnification 10 × 10. **a** Sarcocysts from heart tissue, **b** Sarcocysts from oesophagus tissue

Microscopic sarcocysts were detected in all of the examined fallow deer under light microscope examination in wet mounts. This preliminary microscopic observation revealed the presence of at least three morphs among the isolated sarcocysts depending on their size or shape (Fig. 1a, b).

Two fragments of 18S rDNA (851 bp and 776 bp) were amplified using primers specific for *Sarococystis* spp. The two nucleotide sequences obtained from sarcocysts isolated from the heart were deposited in GenBank under accession numbers KX364266.1 and KX364267.1. A BLAST-N search found that one nucleotide sequence deposited under accession number KX364266.1 share 99.41% similarity with sequences deposited in GenBank under accession numbers MK790239, MK790238, KY973379.1, KY973375.1 and KY973374.1 and described as *S. morae*. Second nucleotide sequence deposited under accession number KX364267.1 share 99.74% identity with sequences KY973379.1, KY973375, MK790239 and MK790238—originally described from red deer and newly described in fallow deer.

The primers specific for *Sarococystis* spp. used in the second PCR protocol were then used to amplify ssu rRNA fragments about 1 kb in length. Three nucleotide sequences of the ssu rRNA were obtained from three sarcocysts: one isolated from fallow deer heart tissue and two isolated from oesophagus tissue. All three sequences were deposited in GenBank under accession numbers MH221019.1 (1064 bp), MH221020.1 (1016 bp) and MH221021.1 (807 bp).

BLAST-N search found nucleotide sequence MH221019.1 obtained from the heart tissue to share 99.91% identity with those from the diaphragm of *Capreolus capreolus* and deposited in GenBank as *S. gracilis* under accession numbers KY019031.1 and KY019030.1*.* Of the nucleotide sequences isolated from the oesophagus, the first (MH221020.1) demonstrated 100% similarity with sequences KY019031.1 and JN256131.1, previously isolated from the diaphragm of *Capreolus capreolus* and described as *S. gracilis*. The second sequence (MH221021.1.) represent *Sarcocystis* sp. and had 96.58%-99.50% identity with *S. linearis* and 97.09–99.01% identity with *S. taeniata*.

**Fig. 2:**
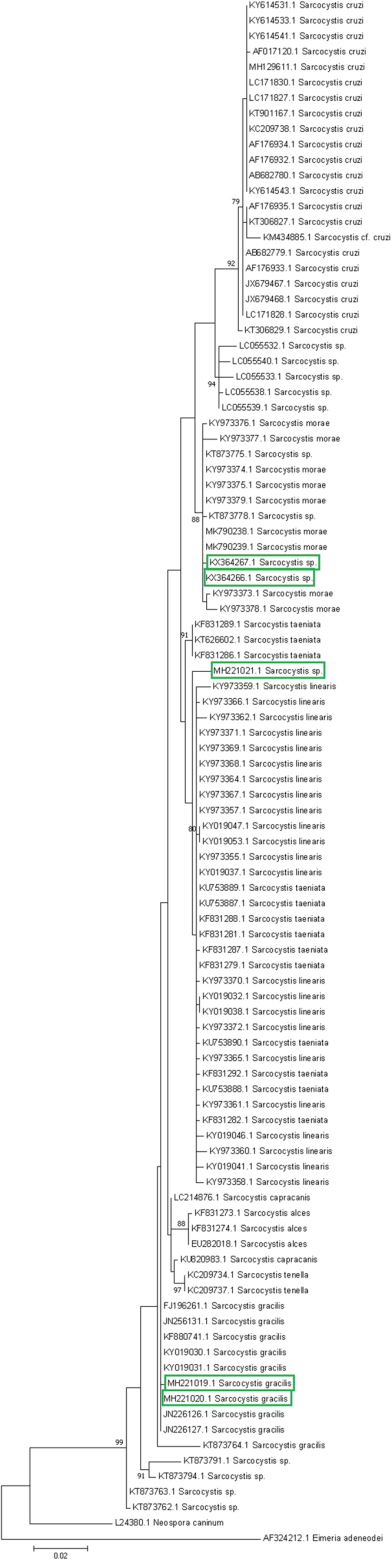
Phylogenetic tree of Sarcocystidae based on ssu rRNA gene sequences and inferred using the maximum likelihood (ML) method. The ssu rRNA tree was constructed based on the alignment of nearly complete ssu rRNA gene sequences of five Polish *Sarcocystis* spp. isolates and available ssu rRNA gene sequences of related species deposited in GenBank. The trees were rooted with *Eimeria adeneodei* and *Neospora caninum* ssu rRNA sequences. Polish isolates are marked in green (color figure online)

The phylogenetic analysis placed two new sequences, MH221019.1 and MH221020.1, within the same clade as *S. gracilis* sequences KY019031.1, KY019030.1, KF880741.1, JN256131.1 and JN226126.1 (Fig. 2). Sequence *Sarcocystis* sp. (MH221021.1) is closely related to *S. linearis* (KY973359.1) and *S. taeniata* (KF831286.1). Further investigations using *cox1* are needed for the conclusive identification of MH221021.1. Sequences KX364266.1 and KX364267.1 were placed in the same clade as *Sarcocystis* sp. sequence KT873778.1 and *S. morae* sequences (MK790239.1 and MK790238.1) both isolated form sarcocysts from the tongue of fallow deer as well as KY973373.1 and KY973378.1 sequences: these were identified from bradyzoites isolated from the oesophagus of *Cervus elaphus*.

## Discussion

Over the last decades, an increasing amount of research has been devoted to the life cycle, morphology and pathogenicity of *Sarcocystis* species. Although *Sarcocystis* infection is common in many domestic and wild animal species, little is known about its occurrence in Poland, with only a handful of studies having been performed. One study based on trichinoscopy and the compression method identified the presence of large-scale invasions of cysts in muscle tissue among 166 wild boars, 53 deer and 53 roe deer in the years 1997–99 [2]. A later study of four Polish roe deer based on light microscopy and sequence analysis of small subunit ribosomal RNA (ssu rRNA) and subunit I of cytochrome oxidase (cox 1) identified the presence of *S. gracilis, S. oviformis* and *S. silva* sarcocysts [24]. *S. tenella* has been detected in Tatra chamois (*Rupicapra rupicapra tatrica*) previously only reported in sheep [25]. A light microscopy study by Pyziel and Demiaszkiewicz (2009) [26] identified *S. cruzi* in heart, oesophagus and diaphragm muscle from one European bison from the Białowieża Forest.

Although *Sarcocystis* spp. have been identified in fallow deer in previous studies [13, 14, 17], the precise species was unknown. Only Hernandez-Rodriquez et al. (1992) [15] described *S. jorrini* sp. nov. as a new species in fallow deer: macroscopic cysts were detected in the skin, and virtually all striated muscles, including those of the esophagus and heart. The cysts themselves were white and spindle shaped, and could clearly be distinguished from the surrounding muscle tissue. Wesemeier and Sedlaczek (1995a) [16] describe the transmission electron microscope identification of sarcocysts in two free-ranging indigenous fallow deer, *Dama d. dama* (L.), from northeast Germany, and one in captive Persian fallow deer (*Dama dama* mesopotamica) from the Berlin-Friedrichsfelde Zoo. In the free-ranging *D. d. dama*, two *Sarcocystis* species were found to be present: one known and the other new to the host. The known species was also found in *D. dama mesopotamica*. Based on a subsequent review of previous studies and their own research on roe deer, red deer and wapiti, Wesemeier and Sedlaczek [27], propose that the two *Sarcocystis* species should be designated *S. cf. grueneri* (known in *D. dama* but not named) and *S. cf. hofmanni* (new for *D. dama*).

Our results confirm the presence of *Sarcocystis* spp. in all tested young fallow deer and suggest that a single year of stay in the pastures was sufficient for the animals to become infected. It is important to note that identified *Sarcocystis* species (*S. morae, S. gracilis* and *Sarcocystis* sp*.*) have not previously been reported in fallow deer in Poland. Lately, *S. morae* was reported in fallow deer as a new host in Spain by De las Cuevas et al. [19]. *Sarcocystis gracilis* is considered to be parasites of roe deer (*Capreolus capreolus*) [28] while *S. morae* occur in red deer (*Cervus elaphus*) [8] and fallow deer (*Dama dama*) [19]. The *Sarcocystis* isolates obtained in the present study shared 96.58–100% identity with those taken previously from different tissues of various hosts from other geographical localities.

Although *Sarcocystis* spp. can be recognized using simple and inexpensive methods, such as naked eye examination or light microscopy, a variety of molecular methods have been developed and implemented to identify *Sarcocystis* spp. and to assess the genetic diversity among these parasite species. The slowly-evolving small subunit (ssu) rRNA gene, commonly used in phylogenetic studies, is also well suited for studying the phylogeny of *Sarcocystis* species [21, 24, 29, 30]. The phylogenetic tree created from the *S. gracilis, S. linearis* and *Sarcocystis* sp*.* sequences from the fallow deer in the present analysis is in agreement with those described in other studies. Two new *S. gracilis* sequences (MH221019.1 and MH221020.1), two new *S. morae* sequences (KX364266.1 and KX364267.1) and one *Sarcocystis* sp. sequence (MH221021.1) were identified and positioned within clades corresponding to other sequences of the same species. BLAST searches and phylogenetic analyses based on ssu rRNA gene sequences confirmed that our isolates closely matched the sequences of those deposited in GenBank and demonstrated a low level of intraspecific sequence variation. Although Januškevičius et al. [31] conclude that no statistically significant differences in infection prevalence exist between muscle groups in the Cervidae, e.g. sika deer, elk, red deer and roe deer, Malakauskas and Grikienienė [32] found infection intensity to be higher in samples of oesophagus and heart muscle than those from the diaphragm. In the present study, samples were only obtained from oesophagus and heart tissue. Therefore, further studies are needed to determine the presence and the intensity of *Sarcocystis* spp. infection in other groups of muscles.

Although approximately 200 species of *Sarcocystis* are recognized, the definitive and intermediate hosts are known for only 62 of them [4]. In the case of *S. gracilis*, the definitive hosts are known to be dogs, red foxes (*Vulpes vulpes*) or blue foxes (*V. lagopus*); the parasite must be consumed by these to complete its life cycle [4, 28]. Domestic dogs, wild dogs and red foxes (*Vulpes vulpes*) are also very common in the region covered by the present study, and these may also serve as definitive hosts; foxes, in particular, have unlimited access to farm animals, unlike dogs. Of the 13 wild carnivorous species known to carry unnamed *Sarcocystis* species [4] four of them, viz*.* raccoons (*Procyon lotor*), European badgers (*Meles meles*), otters (*Lutra lutra*) and mink (*Mustella vision*), are common in the area where the study was carried out.

The definitive hosts of *S. morae* and *S. linearis* are unclear. However, phylogenetic data indicate canids as most likely definitive host of these two species [18, 28]. It is possible that some *Sarcocystis* species in red deer may use other cervids as their principal intermediate hosts, and this may also indicate that many species of *Sarcocystis* are not as specific to their intermediate host as once believed.

## Conclusion

Our study confirms the presence of *Sarcocystis* spp. in farmed fallow deer and is the first to identify three species of the genus *Sarcocystis* in them. Additionally, our results indicate that a wide range of mammals in different areas may harbour *Sarcocystis* species.
